# Reinforcement of reclaimed sand by stimulating native microorganisms for biomineralization

**DOI:** 10.3389/fbioe.2022.1050694

**Published:** 2022-12-22

**Authors:** Shiyu Liu, Yujia Sui, Bowen Dong

**Affiliations:** College of Civil Engineering, Huaqiao University, Xiamen, China

**Keywords:** reclaimed sand, reinforcement, stimulating native microorganisms, biomineralization, mechanism

## Abstract

The method of biological stimulation to reinforce soil has good environmental benefits. The optimization of stimulation solutions can not only improve soil reinforcement but also effectively reduce treatment costs. Response surface methodology was used to optimize a biostimulation solution to reinforce reclaimed sand by native microorganism-induced mineralization. First, response surface methodology was used to obtain the optimal stimulation solution. Then, the effect of the optimal stimulation solution in inducing mineralization to reinforce reclaimed sand was evaluated. Finally, the reinforcement mechanism was revealed by SEM, XRD, and microbial diversity analysis. The results showed that the urease activity of the sample optimized by response surface methodology was 1.17 times higher than that of the sample treated with the initial stimulation solution. The uniaxial compressive strength of samples treated with the optimal biostimulation solution and 1.0 M cementation solution over 15 cycles reached 3.94 MPa. The product of microbial mineralization was calcite, which was the main substance responsible for the improvement in the mechanical properties of the reclaimed sand. The concentration of the cementation solution not only affected the production of calcium carbonate but also affected the morphology of calcium carbonate crystals. After sample treatment with the stimulation solution, ureolytic microorganisms became the dominant bacteria in the sample. A comprehensive assessment of the reinforcement effect and cost revealed that using the optimal stimulation solution and 1.0 M cementation solution over 10 cycles was ideal for reinforcing reclaimed sand. Biostimulation is an effective method to reinforce reclaimed sand; however, the actual application effect requires further examination.

## 1 Introduction

Coastal reclamation, which is one of the most effective methods for ocean-to-land conversion, provides new spaces for urban expansion and industrial and agricultural development. Although this approach poses concerns about ecological problems, it has been applied in the Netherlands, Singapore, Japan and many other countries (Li et al., 2020). Due to the scarcity of land resources in China’s coastal provinces and the strict protection systems currently applied to cultivated land, local governments have adopted coastal reclamation for providing alternative solutions. In recent years, due to the rapid development of China’s economy, the country’s coastal reclamation demand has exceeded 5,880 km^2^, nearly half of the total coastal area reclaimed over the past 50 years (Wei et al., 2015). The main engineering problems encountered during coastal reclamation include site selection, acquisition and transportation of fillings and foundation treatment. Traditional foundation treatment methods, such as compaction, soil mixing and grouting, require considerable amounts of energy and involve high costs. In addition, cement and other chemicals also have adverse effects on the environment (Liu et al., 2021; Rahman et al., 2020; Xue et al., 2021; Wang et al., 2022). Therefore, it is necessary to find an environmentally friendly method of foundation treatment for coastal reclamation.

In recent years, microbially induced carbonate precipitation (MICP) technology has been extensively studied in the field of geotechnical engineering (Cheng et al., 2013; Liu et al., 2020a; Liu et al., 2020b; Cheng et al., 2021; Kim et al., 2021; Xue et al., 2022; Yu et al., 2022; Yu and Rong, 2022). MICP is controllable, green, and non-toxic (Deng et al., 2020). Enzyme-induced carbonate precipitation (EICP) is a bio-cementation technique and a sustainable method of ground improvement (Ahenkorah et al., 2021a; Ahenkorah et al., 2021b). In EICP, calcium carbonate precipitation is produced by urea hydrolysis catalyzed by urease from plants (Almajed et al., 2018; Hu et al., 2021). The smaller size of the urease enzyme making EICP applicable to a wider range of soils (Ahenkorah et al., 2021c) and more uniform distribution of calcium carbonate in soil (Ahenkorah et al., 2020). However, the EICP method lacks nucleation sites for calcium carbonate precipitation (Liu et al., 2021). Microbially induced carbonate precipitation (MICP) can address this limitation of EICP (Liu et al., 2020). Bioaugmentation and biostimulation are the two main treatment strategies for soil improvement by MICP (Liu et al., 2021). Bioaugmentation involves the inoculation of exogenous ureolytic bacteria into the soil. Biostimulation enhances the metabolic activity of native ureolytic microbial communities through nutrient amendment. The ureolysis rates promoted *via* biostimulation are often lower than that of bioaugmentation (Gomez et al., 2017). However, native bacteria may be more adaptable than cultured bacteria to the natural environment (Acea et al., 1988), resulting in more persistent ureolytic activity throughout treatment. In addition, bioaugmentation requires the introduction of exogenous bacteria, which raises environmental concerns (Gomez et al., 2018). Biostimulation does not require bacterial cultures, transportation or grouting, which not only reduces costs but also prevents risks associated with species invasion (Litchman, 2010). Therefore, biostimulation has attracted much attention from researchers. Ravehamit and Tsesarsky, (2020) found that ureolytic bacteria can be effectively activated even in desert soils that contain very low organic matter contents. Wang et al. (2020) found that the addition of a small amount of ammonium to a stimulation solution can increase the urease activity of samples, resulting in improved stimulation. The study of Gomez et al. (2019) showed that the composition and proportion of a stimulation solution had an important effect on the strength of the treated samples. The above studies have proven the effectiveness of biological stimulation methods for reinforcing soil, but no in-depth research exists on the key issue of optimizing stimulation solutions. The optimization of stimulation solutions can not only improve soil reinforcement but also effectively reduce treatment costs.

The purpose of this study is to propose a strategy to optimize biostimulation solutions based on a response surface method and, through a series of experiments, verify the feasibility of using a biostimulation strategy to reinforce reclaimed sand. According to the test results, the effects of cementation solution concentrations and treatment cycles are further analyzed. The samples are also analyzed by scanning electron microscopy (SEM) and X-ray diffraction (XRD) to clarify the reinforcement mechanisms active in reclaimed sand.

## 2 Materials and methods

### 2.1 Materials

Corn steep liquor, soybean meal and starch were purchased from Beijing Hongrun Baoshun Technology Co., Ltd., and molasses was purchased from Guangxi Jianli Chemical Trading Co., Ltd. All other reagents were of analytical grade and were purchased from China National Pharmaceutical Group Corporation.

**FIGURE 1:**
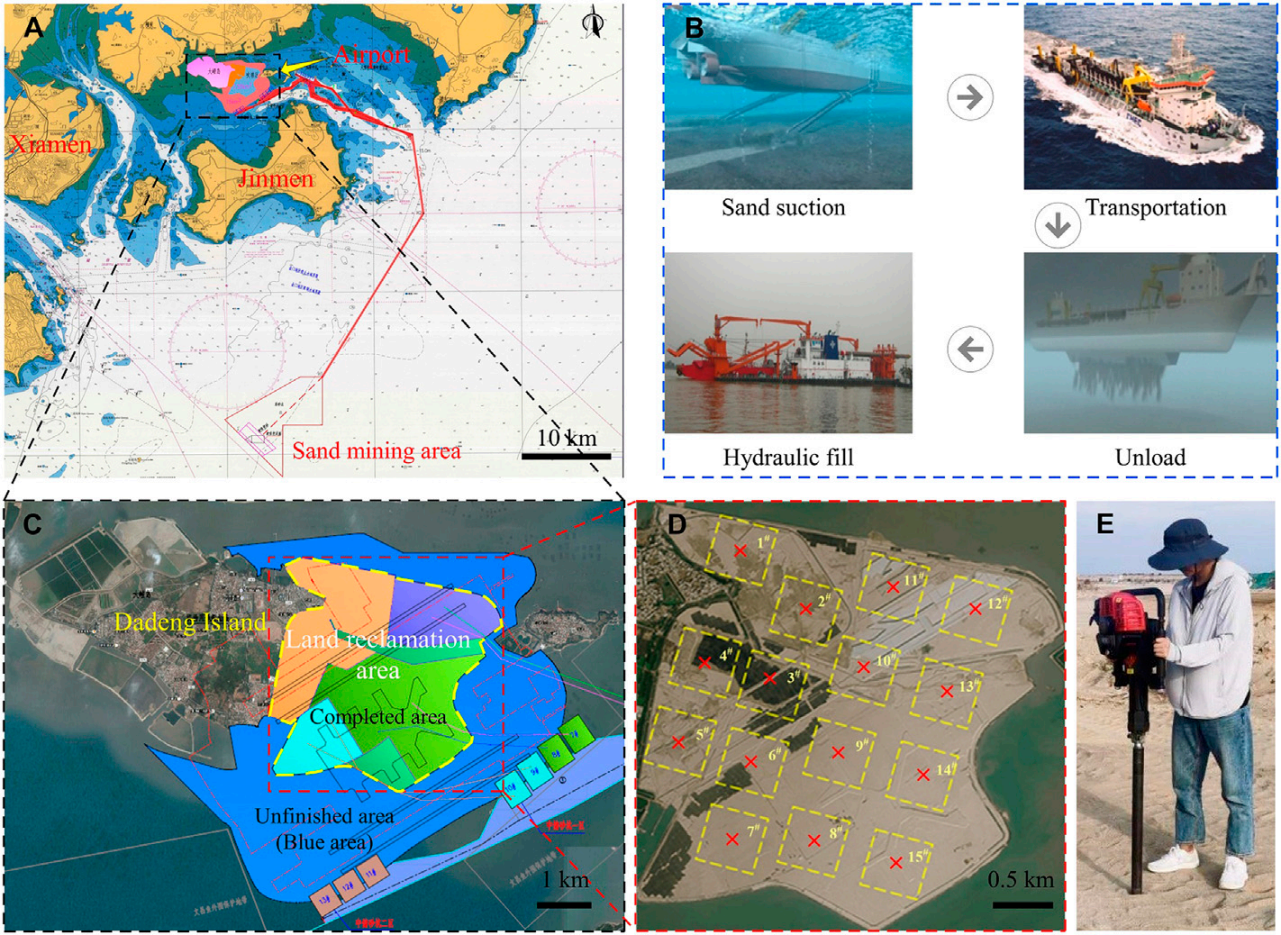
The location of the airport under construction and the sampling diagram: **(A)** location of the airport; **(B)** construction process with sea sand; **(C)** land reclamation area; **(D)** sampling diagram; **(E)** sampling photo.

**FIGURE 2:**
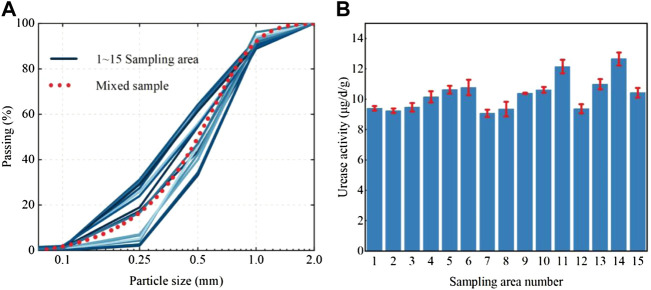
Particle size distribution curve **(A)** and urease activity **(B)** of samples.

The sea sand used in the test was obtained by sampling from the Xiamen Xiang’an International Airport project site. The airport site is shown in Figure 1A, and its construction was carried out by means of land reclamation. The reclamation material was natural sea sand, and the sand mining area was located in a region offshore of Zhangzhou, which is approximately 50 km away from the airport (Figure 1A). The construction process for sea sand is shown in Figure 1B. The average sand filling thickness at the airport was 8.0 m. In the completed reclamation area of about 15 km^2^ (Figure 1C), 15 sampling areas of dimensions 500 m × 500 m were selected (Figure 1D), and a five-point sampling method was used for sampling. Sand from depths between 5 m and 6 m was collected to verify the effect of biostimulation-induced mineralization for strengthening deep soils. Sampling was carried out with a small drilling sampler (Figure 1E), and the tools and containers were sterilized before sampling. The soil parameters were determined according to the Specification of Soil Test (MWR, 1999), and the calculated values are shown in Table 1. According to the parameters, the sand was determined to be poorly graded. The particle size distribution curve of the sand is provided in Figures 2A,B. shows that all samples had urease activity, indicating the existence of ureolytic microorganisms in sea sand, which provided a prerequisite for biostimulation-induced mineralization. The NH_3_-N produced by urease hydrolysis of urea was determined by indophenol blue colorimetric method (Yi et al., 2022), and the blue indophenol produced was proportional to the concentration of ammonia. The production of 1 μg NH_3_-N per g of soil sample per day is defined as a unit of enzyme activity (µg/d/g). The Micro Soil Urease (UE) Assay Kit (Solarbio, China) was used to measure urease activity. Specifically, 20 μL of methylbenzene was added to 0.05 g soil that was sieved through a 0.0425-mm mesh, and the mixture was kept at room temperature for 15 min. Then 90 μL urea solution and 190 μL buffer solution were added and incubated at 37°C for 24 h, followed by centrifugation and coloration. The urease activity was measured spectrophotometrically at 630 nm. Since the sea sand came from the same area, the physical and chemical properties of the sand from each sampling area were not very different, so the sand from each sampling area was mixed for biostimulation. The mineral component of the sand was quartz, which did not react with the stimulation solution and cementation solution.

**TABLE 1:** Physical properties of reclaimed sand.

Soil properties	Value
d10	0.189 mm
d30	0.335 mm
d60	0.582 mm
Coefficient of non-uniformity (Cu)	3.079
Coefficient of curvature (Cc)	1.146
Minimum dry density	1.458 g/cm3
Maximum dry density	1.738 g/cm3
Maximum void ratio	0.674
Minimum void ratio	0.450
Specific gravity, GS	2.590 g/cm3

### 2.2 Biostimulation optimization

Based on the initial stimulation solution, the optimal stimulation solution for sea sand was obtained by a single factor test, by a Plackett-Burman (PB) design and by central combinatorial design (CCD) response surface analysis. The initial stimulation solution was established based on a previous study (Gomez et al., 2018, 2019; Liu et al., 2021). The concentrations of each component in the initial stimulation solution were as follows: sodium acetate (42.5 mM), ammonium chloride (100 mM), urea (333 mM), nickel chloride (0.01 mM), and YE (0.2 g/L), and the pH was 9.0.

Based on the initial stimulation solution, a single factor test was carried out by changing the carbon source or nitrogen source. Glucose, fructose, maltose, starch, and molasses were selected as carbon sources to be evaluated. Ammonium chloride, ammonium sulfate and sodium nitrate were selected from inorganic nitrogen sources, and peptone, corn steep liquor and soybean meal were selected from organic nitrogen sources. First, 10 g air-dried soil was added to a conical flask containing 90 ml stimulation solution, and then the flask was shaken in an incubator (30°C, 180 rpm). According to the method proposed by Whiffin (2004), the urease activity of the sample at different times was obtained by electrical conductivity. A Con700 conductivity meter (Eutech Instruments Pte Ltd., Singapore) was used for measurements. By using urease activity as the evaluation index, the optimal carbon source and nitrogen source were selected.

Based on the single-factor test, the substances exhibiting high urease activities were selected from the carbon sources, organic nitrogen sources and inorganic nitrogen sources for PB design to determine the key factors. After determining the concentration of the key factors and using the urease activity as the response target value, CCD response surface analysis was performed using Design Expert 10 software (Stat-ease Corporation) to obtain the optimal stimulation solution.

### 2.3 Sample preparation and treatment

The collected sand was passed through a 2 mm sieve to remove impurities such as shell debris. Containers and tools were sterilized before sample preparation. The mold used for the test was a PVC tube with a height of 80 mm and an inner diameter of 35 mm. The sand used for filling was divided into three layers and compacted with an aseptic steel rod. The density of the sand column after filling was the maximum dry density of 1.738 g/cm^3^. A fixing bracket was used to secure the two pipe covers at both ends of the PVC tube.

**FIGURE 3:**
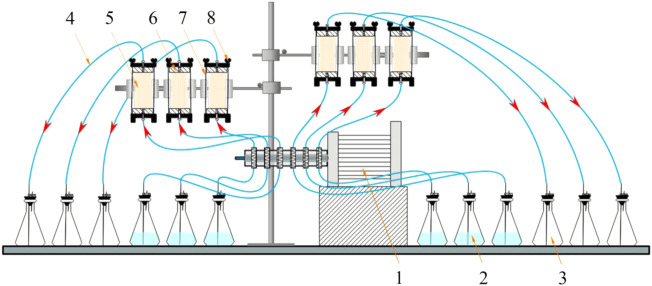
Schematic diagram of grouting: 1. Peristaltic pump; 2. Treatment solution; 3. Waste liquid bottle; 4. Silicone tube; 5. Sand column; 6. Tube cap; 7. Fixed bracket; 8. Butterfly nut.

**FIGURE 4:**
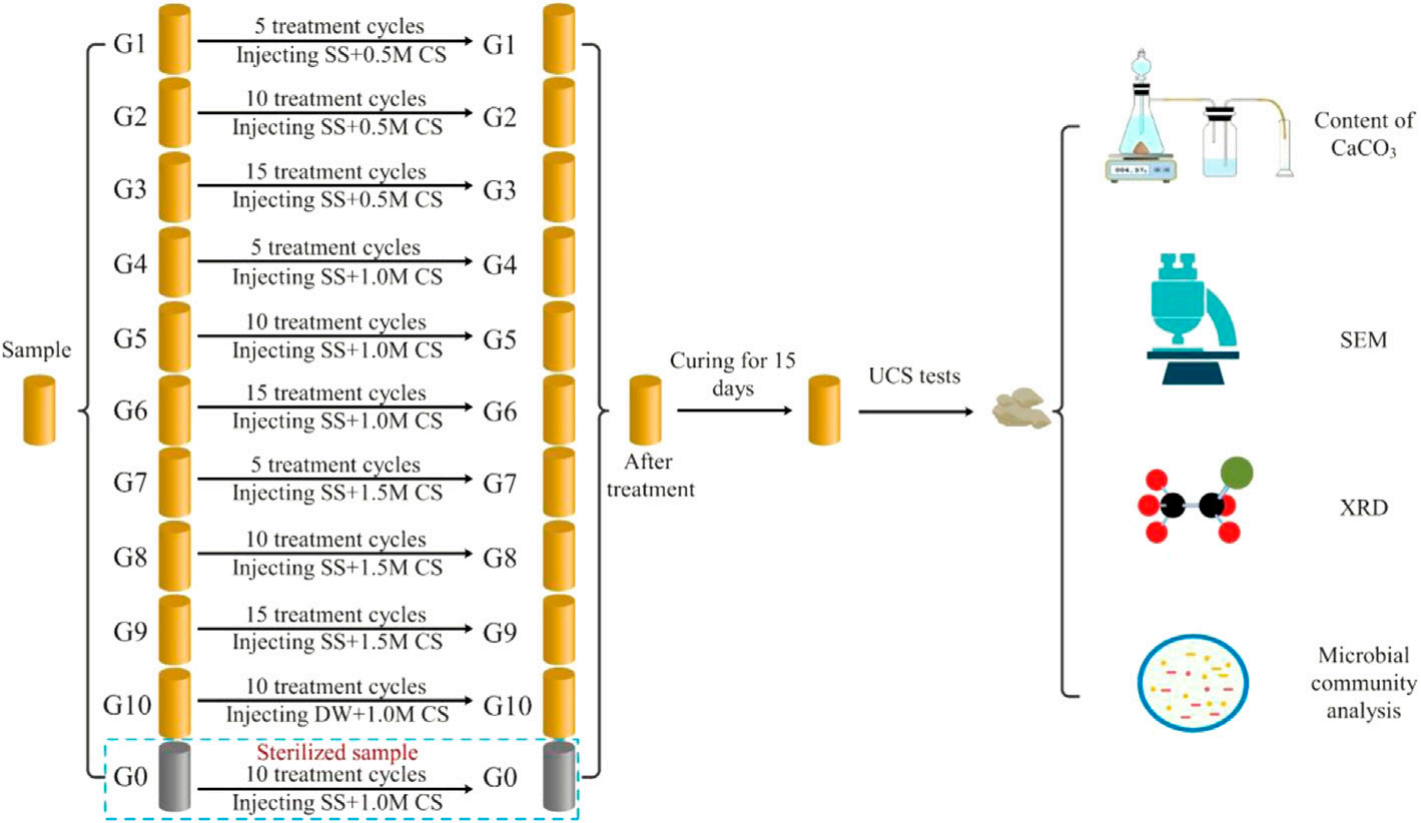
Schematic of the test procedures (SS—stimulation solution; CS—cementation solution; DW—deionized water).

After sample preparation was complete, 50 ml of stimulation solution (1.5 PV) was first injected, and then 50 ml of cementation solution (equimolar concentrations of urea and calcium chloride) was injected after 72 h. The number of treatment cycles was 5, 10, and 15, and the interval between each cycle was 24 h. Deionized water was used to replace the stimulation solution in the control group (G10), and the G0 group was the sterile sample. The treatment solution was injected into the sample from the bottom of the column using a peristaltic pump at 1 ml/min (as shown in Figure 3). All solutions were filter-sterilized using 0.22 μm vacuum filters (Guangzhou Jet Bio-Filtration Co., Ltd., China) to avoid the influence of miscellaneous bacteria. According to the research of Cheng et al. (2021), the samples were treated with MICP and left standing for 15 days to complete the mineralization reaction. After 15 days of treatment, the sand columns were washed with deionized water to remove soluble salts and residual organic matter. Then, the mold was removed, and the sand column was dried to constant weight. In line with past studies (Whiffin, 2004), treated specimens were oven-dried at 60°C before testing. This approach does not reflect a field condition and caution should be applied for direct translation of knowledge (Ahenkorah et al., 2020). The sample processing scheme is shown in Figure 4.

### 2.4 UCS tests

UCS testing was carried out using a TSZ-3 strain control triaxial apparatus from Nanjing Soil Instrument Factory Co., Ltd., China, according to the Specification of Soil Test (MWR, 1999). A loading rate of 0.8 mm/min was used until the sample was destroyed. A total of 33 samples (11 groups, 3 parallel samples per group) were prepared for UCS testing. The height of the sample was 7.0 cm and the diameter was 3.5 cm (the ratio of height to diameter was 2:1). Grinded the bottom and top of the sample to make sure it was flat before UCS testing. The strain in the UCS test was calculated by dividing the deformation by the initial length of the sample. The stress in the UCS test was calculated as *σ* = 4*F*/*πD* ^2^, where σ is the compressive stress; *F* is the vertical load; and *D* is the diameter of the specimen.

### 2.5 Determination of CaCO_3_ content

After the UCS test, the content of CaCO_3_ in the sample was measured by a washing method (Choi et al., 2017). The sample was washed with ultrapure water 3 times before the test and washed with 1.0 M HCl after drying. The amount of CaCO_3_ was determined according to the change in sample mass before and after acid washing.

### 2.6 Microscale identification analysis

SEM analysis was carried out with a SU1510 scanning electron microscope produced by Hitachi Ltd., Japan. The accelerated voltage was 5 or 10 kV. The samples were treated with gold spray before observation. XRD analysis was conducted using a Smart Lab X-ray diffractometer (Rigaku Corporation, Japan) with 40 kV and 40 mA CuKα radiation, and the XRD patterns were obtained at a scanning rate of 10°/min from 4° to 90° 2θ.

### 2.7 Microbial community analysis

Total deoxyribonucleic acid (DNA) was extracted from untreated sand and treated sand using an E.Z.N.A. extraction Mag-Bind Soil DNA Kit made by the OMEGA Company. The final sequencing process was carried out according to the guidelines for the preparation of the Illumina 16 S ROD database sequencing library of Shanghai Sangon Biotechnology Co., Ltd. According to the sequencing results, the abundance and diversity of the microbial community before and after sample treatment were analyzed.

## 3 Results and discussion

### 3.1 The optimal stimulation solution

#### 3.1.1 Single-factor test

**FIGURE 5:**
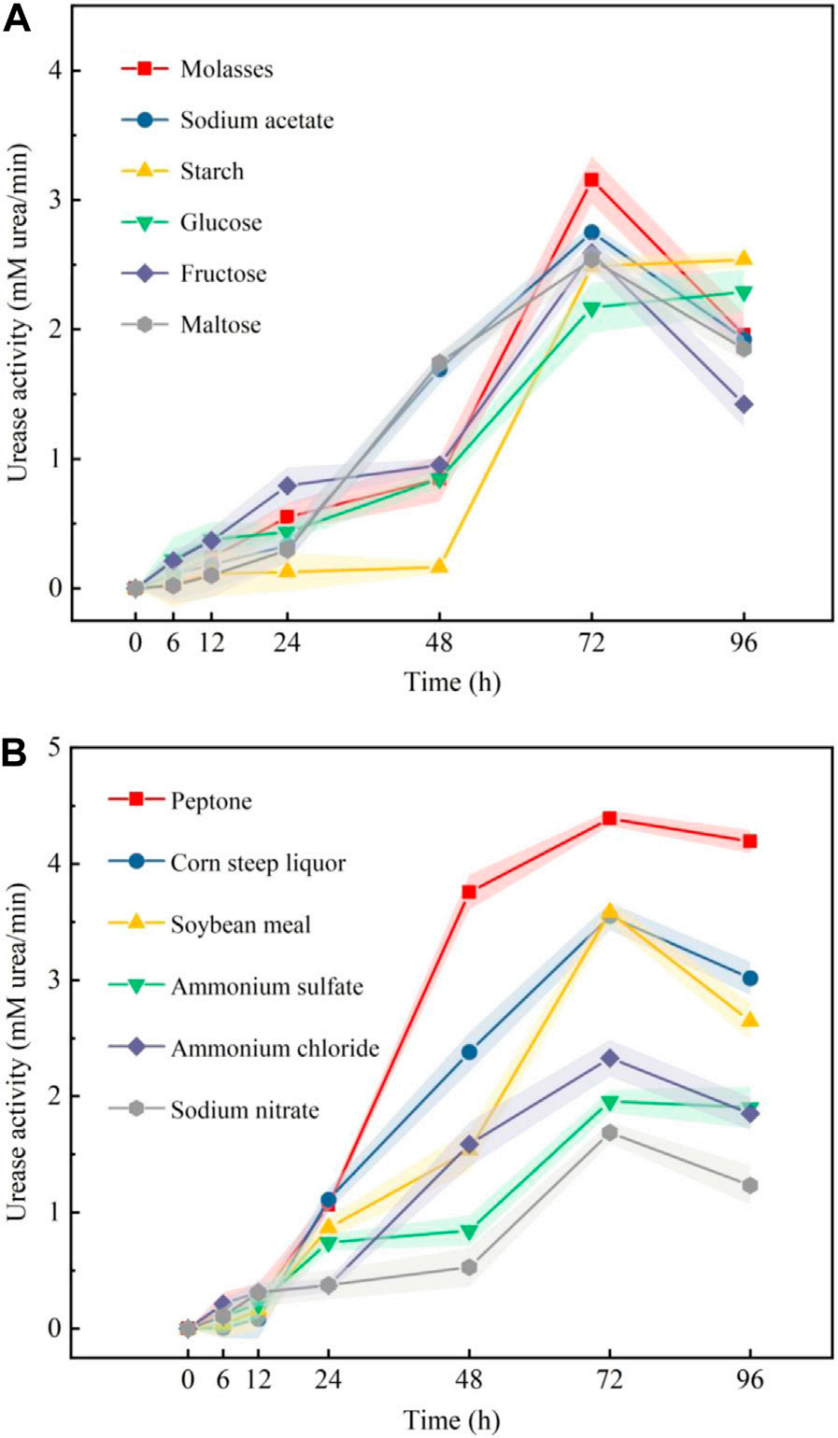
Effect of different carbon sources **(A)** and nitrogen sources **(B)** on the urease activity of samples.

The changes in urease activity that occurred with time using different carbon sources and nitrogen sources are shown in Figure 5A and Figure 5B, respectively. Figure 5A shows that molasses and sodium acetate induced higher urease activities than the other carbon sources. Figure 5B shows that the urease activities induced by ammonium chloride and ammonium sulfate were higher than those of other inorganic nitrogen sources. The urease activities induced by peptone and corn steep liquor were high among organic nitrogen sources. According to the test results shown in Figures 5A,B, molasses, sodium acetate, ammonium chloride, ammonium sulfate, peptone, and corn steep liquor were selected for PB design.

#### 3.1.2 Results of PB design

The PB design and experimental results based on the single-factor test are shown in Table 2. The analysis results showed that the *p* values of molasses, ammonium chloride, nickel chloride, urea, YE and pH were all less than 0.05, indicating that the above components were significant factors that affected urease activity. The components of the stimulation solution were divided into four parts: carbon source, nitrogen source, growth factor and environmental factor. According to the above analysis results, molasses, ammonium chloride, nickel chloride and pH were selected from these four parts as the key factors affecting urease activity.

**TABLE 2:** Results of PB design.

Factor	Level		F Value	*p*-value	Estimated coefficient
	−1	+1			
Molasses (g/L)	3.5	5.2	167.36	0.049	0.480
Sodium acetate (mM)	42.50	63.75	67.03	0.077	0.300
Peptone (g/L)	5	7.5	133.42	0.055	−0.390
Corn steep liquor (g/L)	5	7.5	53.92	0.086	0.230
Ammonium chloride (mM)	100	150	383.47	0.032	−0.760
Ammonium sulfate (mM)	37.83	56.75	1.99	0.393	−0.015
Nickel chloride (mM)	0.010	0.015	215.22	0.043	−0.550
Urea (mM)	333	500	194.17	0.046	−0.660
YE (g/L)	0.2	0.3	357.75	0.034	0.720
PH	7.0	9.0	342.54	0.034	0.110

**FIGURE 6:**
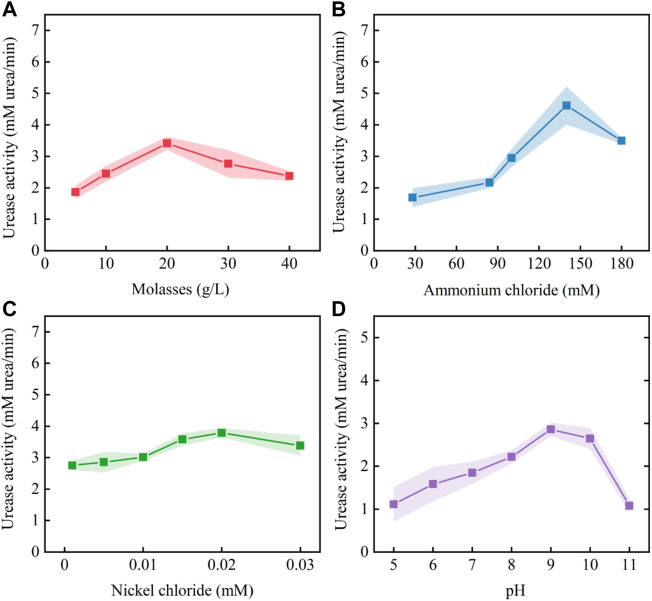
Effect of each key factor on urease activity: **(A)** molasses; **(B)** ammonium chloride; **(C)** nickel chloride; and **(D)** pH.

According to the estimated positive and negative coefficients shown in Table 2, the initial stimulation solution was adjusted to molasses (5.2 g/L), ammonium chloride (100 mM), nickel chloride (0.01 mM), urea (333 mM), Ye (0.2 g/L) and pH = 9.0. Based on the adjusted stimulation solution, a single-factor test was carried out to determine the optimal concentration ranges for the key factors. The test results (Figure 6) show that the optimum concentration ranges for molasses, ammonium chloride and nickel chloride were 10–30 g/L, 100–180 mM and 0.01–0.03 mM, respectively. The optimum range of pH was 8–10.

#### 3.1.3 CCD response surface analysis

The CCD design is shown in Table 3, and the shaking flask test was carried out according to the design. The test results were analyzed by Design Expert 10 software, and the quadratic polynomial regression model was constructed. The analysis of variance of the model is shown in Table 4. The *p*-value of the regression model was very small, indicating that the model was extremely significant. The *R* ^*2*^ value was 96.62%, indicating that the prediction results of the model were reliable.

**TABLE 3:** Factors and levels of the CCD test.

Factor	Level				
	−2	−1	0	1	2
Molasses (g/L)	10	15	20	25	30
Ammonium chloride (mM)	100	120	140	160	180
Nickel chloride (mM)	0.010	0.015	0.020	0.025	0.030
PH	8	8.5	9	9.5	10

**TABLE 4:** Analysis of variance of the response surface model.

Source	df	Sum of squares	Mean square	*F* Value	*p*-value Prob > *F*
Model	14	39.730	2.840	30.66	<0.0001
A	1	0.650	0.650	7.00	0.0183
B	1	0.320	0.320	3.41	0.0844
C	1	0.082	0.082	0.89	0.3611
D	1	0.640	0.640	6.91	0.0190
A^2^	1	9.020	9.020	97.41	<0.0001
B^2^	1	18.490	18.490	199.73	<0.0001
C^2^	1	2.590	2.590	28.01	<0.0001
D^2^	1	12.40	12.400	134.01	<0.0001
AB	1	0.050	0.050	0.54	0.4739
AC	1	0.290	0.290	3.08	0.0996
AD	1	0.950	0.950	10.24	0.0060
BC	1	1.860	1.860	20.10	0.0004
BD	1	1.030	1.030	11.08	0.0046
CD	1	2.330	2.330	25.19	0.0002
Pure Error	5	0.180			
Cor Total	29	41.120			
*R* ^2^ = 96.62%		*R* ^2^ (adj.) = 93.47%		*C.V.*% = 6.35	

Note: The concentration values of sodium acetate, corn steep liquor, YE, to be A, B, C, and pH is D.

**FIGURE 7:**
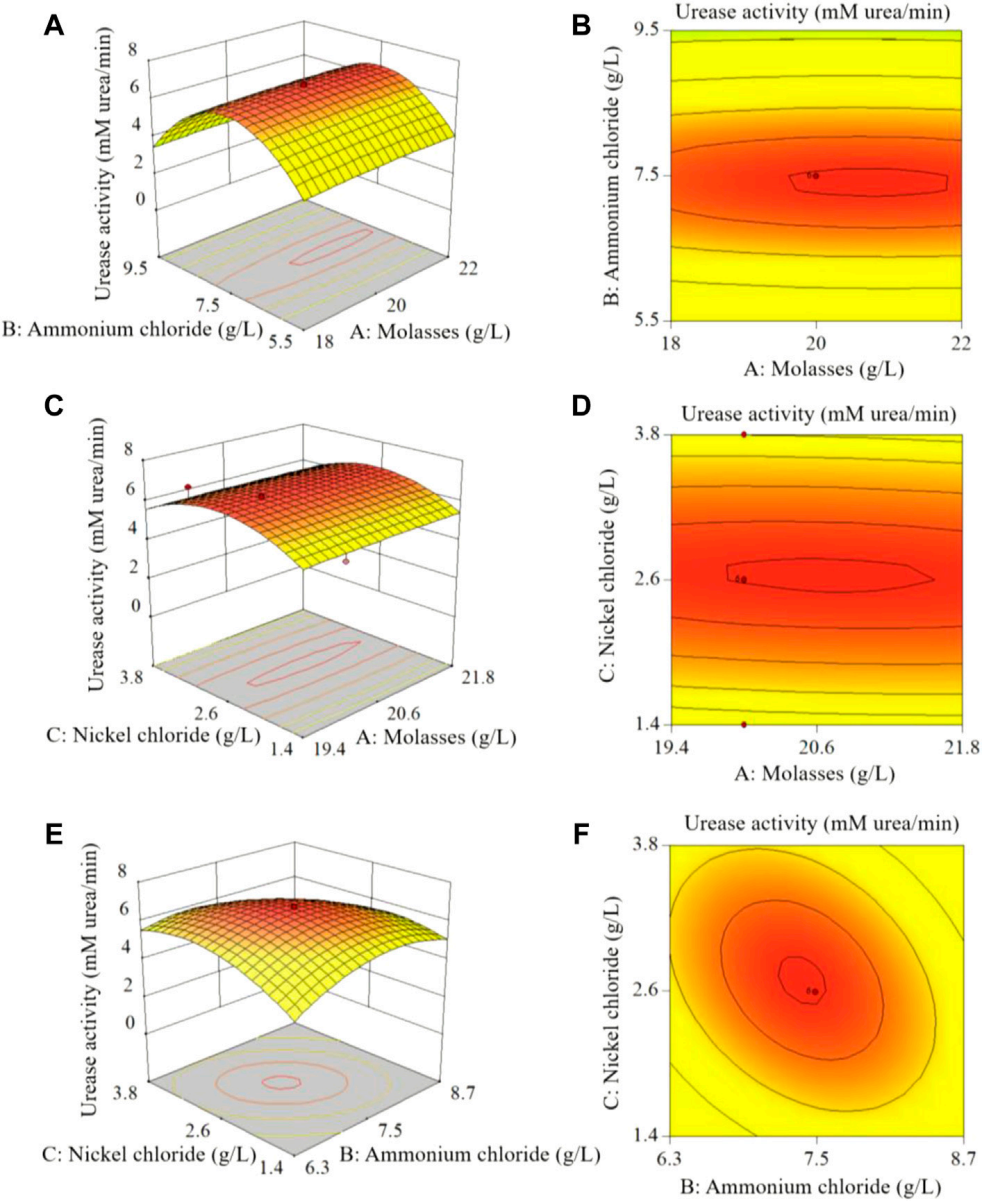
Analysis of CCD test results: **(A)**, **(C)**, **(E)** show the 3D response surface plots of the effects of molasses, ammonium chloride and nickel chloride concentrations on urease activity; **(B)**, **(D)**, **(F)** are the horizontal projections of the **(A)**, **(C)**, **(E)** 3D plots, respectively.

Figure 7 shows the effects of molasses, ammonium chloride and nickel chloride concentrations on urease activity. As shown in Figure 7B, when the concentration of nickel chloride was 0.02 mM, the curves for the effect of molasses and ammonium chloride on urease activity were elliptical, and the isoline change along the direction of ammonium chloride concentration was dense. This shows that there was an interaction between molasses and ammonium chloride, and the effect of ammonium chloride on urease activity was greater than that of molasses. Similarly, Figures 7D, F show that the effect of nickel chloride on urease activity was greater than that of molasses and ammonium chloride.

The results of the CCD test showed that the concentrations of molasses, ammonium chloride, nickel chloride, urea and YE in the optimal stimulation solution were 23.21 g/L, 135.48, 0.02, 333 mM and 0.2 g/L, respectively, and the pH value was 8.64. The corresponding urease activity was 6.52 mM urea/min, which was 1.17 times higher than that of the initial stimulation solution (3.01 mM urea/min). Molasses is a cheap carbon source containing glucose, fructose, and sucrose that can stimulate a wide range of bacteria (Kiasari et al., 2019). Based on the above reasons, scholars use molasses to stimulate the growth of ureolytic microorganisms (Tobler et al., 2011; Gat et al., 2016; Kiasari et al., 2019; Liu et al., 2021), and the results confirm the effectiveness of molasses. Moreover, as molasses does not provide biologically available forms of nitrogen, it is less likely to affect urease expression in ureolytic bacteria exhibiting nitrogen-concentration dependent regulation (Burbank et al., 2011). Using molasses as a cost-effective alternative to YE for *in situ* applications of MICP might prevent eutrophication and a complete consumption of oxygen (Gat et al., 2016).

### 3.2 UCS test and analysis

**FIGURE 8:**
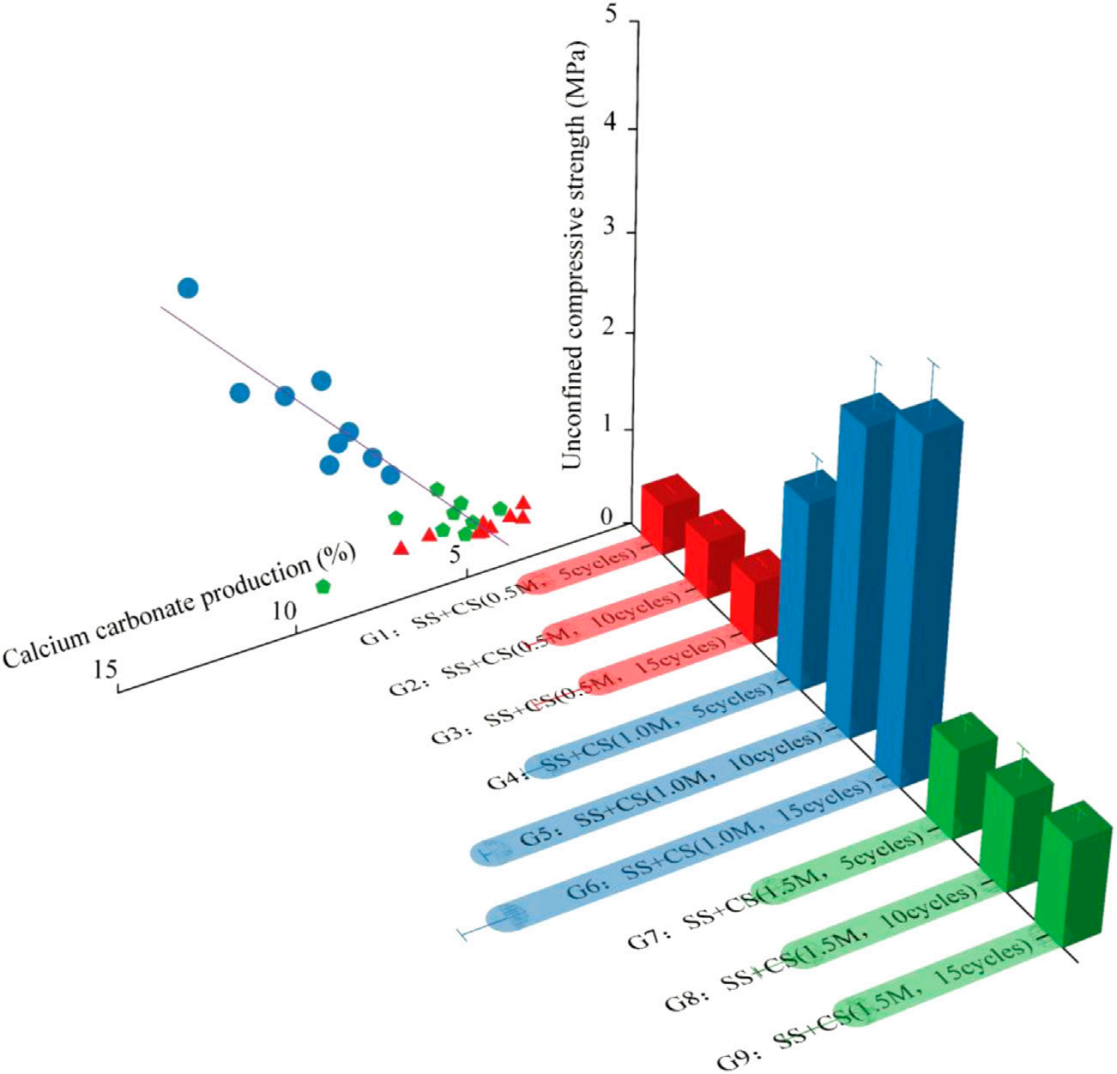
Relationship between calcium carbonate content and uniaxial compressive strength.

Figure 8 shows the UCS of samples with different treatment methods. Figure 8 shows that the strength of the sterilized sample (G0) was 0 kPa, indicating that the presence of bacteria is a prerequisite for using biostimulation to induce mineralization. The strength of the control sample (G10) was also 0 kPa, suggesting that the strength of the sample cannot be improved by using a cementation solution alone without the injection of a stimulation solution. However, the samples treated with the biostimulation treatment achieved a certain improvement in strength. The strength of the samples treated with 1.0 M cementation solution over 10 and 15 cycles reached 3.54 and 3.94 MPa, respectively.

It is worth noting that from the UCS results, it was found that the UCS of the sample decreased when the concentration of cement solution was 1.5 M. This is because a high cementation solution concentration can accelerate the precipitation of calcium carbonate (Al Qabany and Soga, 2013; Chu et al., 2014; Cao et al., 2022), which is easily impeded near the grouting port, so the distribution of calcium carbonate in the sample was not uniform. In addition, excessively high calcium concentrations can reduce the urease activity of bacteria (Kunst and Rapoport, 1995). The reason may be that a high concentration of CaCl_2_ has a high osmotic pressure, which can directly or indirectly affect the metabolic activity and survival rate of bacteria (Liu et al., 2020b). A comprehensive consideration of the reinforcement effect and cost shows that using the optimal stimulation solution and 1.0 M cementation solution for 10 cycles is an ideal choice for reinforcing reclaimed sand.

### 3.3 Calcium carbonate content

The amount of calcium carbonate produced in each group of samples after treatment is shown in Figure 8. The amount of calcium carbonate produced in the sterilized group (G0) was zero, which corresponded to the UCS results. The amount of calcium carbonate produced in the control group (G10) was less than 0.5%, which may have resulted from mineralization induced by a small number of ureolytic microorganisms present in the reclaimed sand. However, the calcium carbonate content of the samples treated with the biostimulation solution was significantly higher. The calcium carbonate content of the samples treated with the same concentration of cementation solution increased with an increase in the number of treatment cycles. For the same treatment cycle, the amount of calcium carbonate first increased and then decreased with an increase in the concentration of the cementation solution. This trend was the same as that of the UCS test results, indicating that the amount of calcium carbonate produced affects the UCS of samples.

Figure 8 shows the relationship between the UCS and calcium carbonate content of the biostimulated samples. The figure shows that there is a linear correlation between UCS and calcium carbonate content. The results of this study are consistent with those of Liu et al. (2021) and Dagliya et al. (2021). This result indicated that the calcium carbonate generated by stimulating native microorganism-induced mineralization was the main reason for the improvement in the mechanical properties of the samples. The reversing of amendments was done to achieve more uniform calcite precipitation by avoiding plugging near the injection source (Martinez et al., 2013; Nafisi et al., 2019; Ahenkorah et al., 2020). However, this study only verifies the feasibility of reinforcing reclaimed sand by biological stimulation. Therefore, according to the research of Gomez et al. (2018), the samples were treated by unidirectional grouting (injecting the treatment solution from the bottom of the sample), but the reverse grouting was not carried out to improve the uniformity of calcium carbonate distribution.

### 3.4 SEM test results and analysis

**FIGURE 9:**
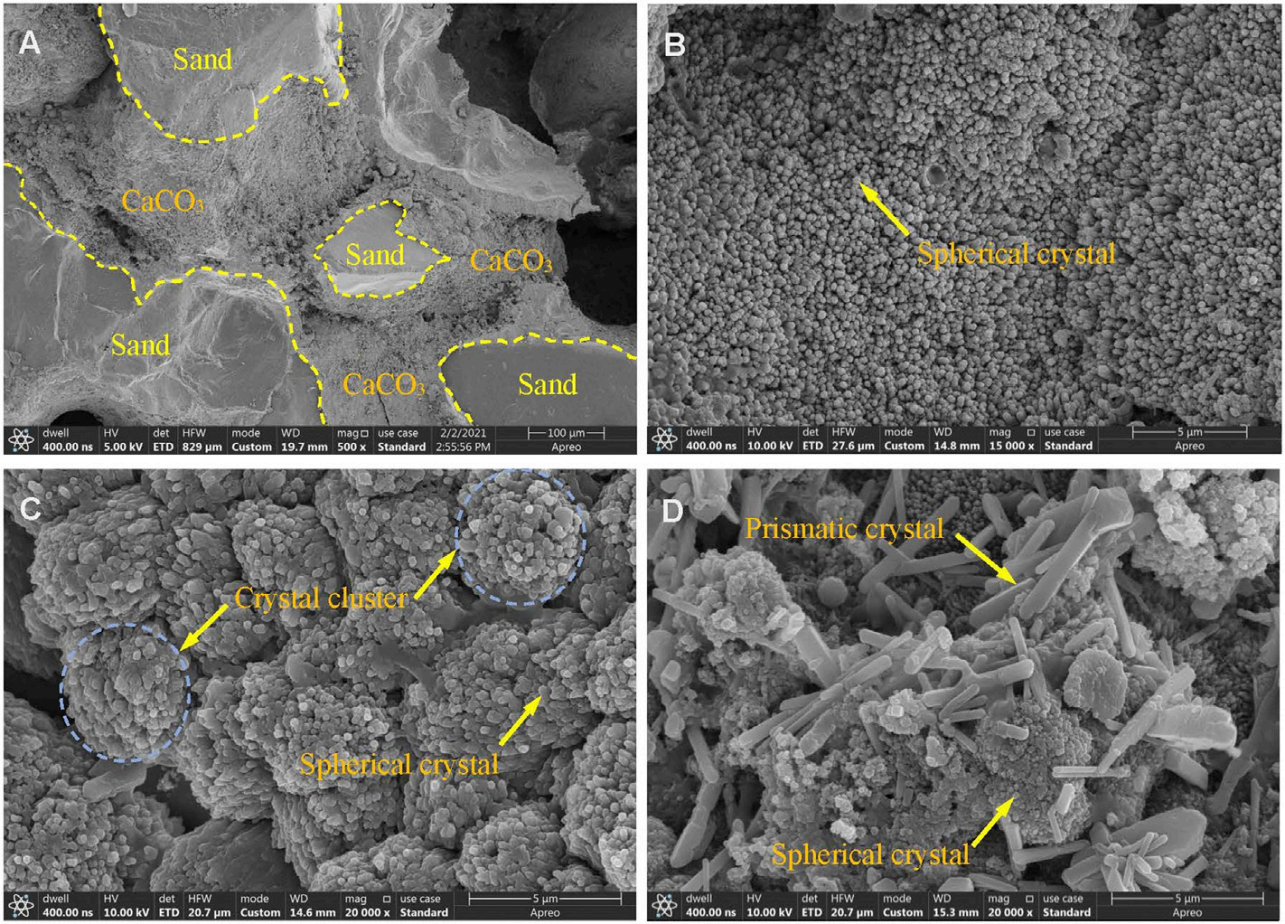
SEM of treated samples: **(A)**, **(B)**, **(C)**, and **(D)** are the results of samples G5, G3, G6, and G9, respectively.

Figure 9 shows the SEM test results of the samples, (a) shows a photo of sample G5, and (b), (c) and (d) are photos of samples G3, G6, and G9, respectively. As shown in Figure 9A, after treatment with the biostimulation strategy, the surfaces of the sand particles were covered by the calcium carbonate generated during treatment, and the pores between the sand particles were filled with the mineralized products. The crystal particles shown in Figures 9B,C were mainly spherical, but the spherical, mineralized particles produced were more abundant in Figure 9C and formed crystal clusters. The morphology of the mineralized particles produced (Figure 9D) was diverse and included spherical and prismatic shapes, and the prismatic crystals were relatively large in size. The different concentrations of the cementation solution used during the treatment process were the main reason for the different morphologies of the mineralized products in Figures 9B–D.

The nucleation of new crystals competes with crystal growth during mineralization (Gandhi et al., 1995). The low extracellular calcium and carbonate concentration in soil pore fluid is beneficial for now calcium carbonate crystal nucleation, while high extracellular calcium and carbonate concentration in pore fluid is beneficial for calcium carbonate crystal growth (Somani et al., 2006; Jiang et al., 2019). Snoeyink and Jenkins (1980) stated that the necessary degree of supersaturation for precipitation tends to be larger for homogeneous nucleation (i.e., growth of calcite crystals) than for heterogeneous nucleation (i.e., nucleation over sand grains). Such high supersaturation may also be a result of organics produced by bacteria [such as extracellular polymeric substances (EPS)] acting as crystallization inhibitors (Rodriguez-Navarro et al., 2007). In this study, when the concentration of the cementation solution was less than 1.0 M, the concentration of extracellular calcium and carbonate in the sand pore fluid was beneficial for the nucleation of calcium carbonate crystals, so the crystals in Figures 9B,C were mainly spherical. When the concentration of the cementation solution was 1.5 M, the concentration of extracellular calcium and carbonate in the sand pore fluid was beneficial for the growth of calcium carbonate crystals. Therefore, larger crystal particles appeared in Figure 9D, and the prismatic crystals of different sizes present in the figure also showed that the growth process proceeded from small crystals to large crystals. The crystal size of calcium carbonate formed by higher concentration of cementing solution is larger. However, it can be seen from Figure 8 that the strength of the sand column is related to the amount of calcium carbonate, but does not seem to be directly related to the crystal size. Jiang et al. (2019) research also found a similar phenomenon.

### 3.5 X-ray diffraction test results and analysis

**FIGURE 10:**
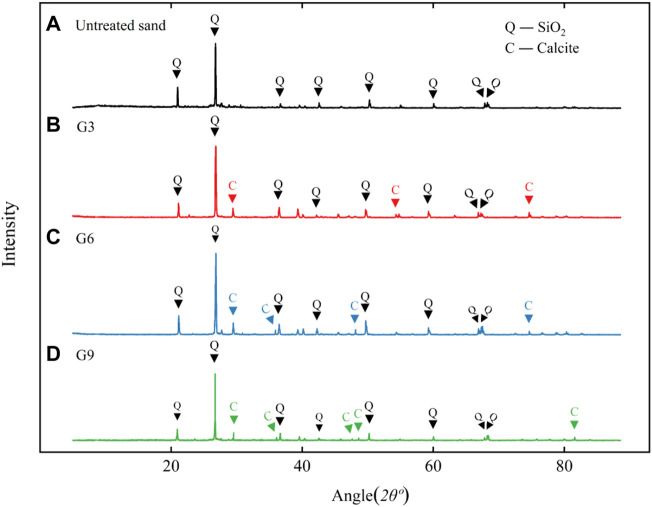
XRD analysis results of treated samples: **(A)**, **(B)**, **(C)**, and **(D)** are the results for untreated sand, G3, G6, and G9, respectively.

The X-ray diffraction test results of the treated and untreated samples are shown in Figure 10. The main component of untreated reclaimed sand was silica, while high absorption peaks for calcite appeared in the samples treated with biological stimulation. This shows that calcite was the product of mineralization induced by biological stimulation. The crystal forms of calcium carbonate are mainly vaterite, aragonite and calcite, and calcite is relatively stable (Gomez et al., 2018).

### 3.6 Results of microbial community analysis

**FIGURE 11:**
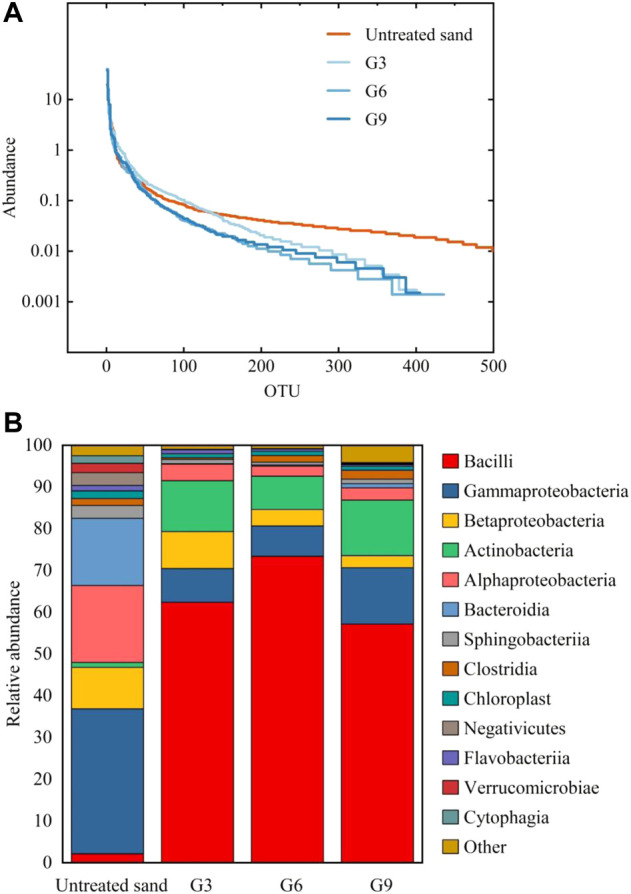
Rank-abundance curve **(A)** and relative abundance of microorganisms **(B)** in different samples.

Figure 11A shows the rank-abundance curve of the samples. The curve for the untreated sand covered more OTU types and was flatter, indicating that the microbial abundance in untreated sand was high and the species composition was relatively uniform, whereas the curve for the biostimulation-treated samples was shorter and ladder-like, indicating that the species abundance was low and uneven. Biostimulation solutions create a selective environment, and some microorganisms that are unsuited to this environment do not survive, so the number of species and abundance of microorganisms decrease.

Figure 11B shows the relative abundance of microorganisms in different samples at the class level. The comparison shows that after biostimulation treatment, the dominant species of microorganisms changed significantly, and *Bacillus* replaced *Proteobacteria* to become the dominant bacteria. Bacillus is the dominant bacteria responsible for urea decomposition and includes species such as *Bacillus Pasteurellosis* and *Bacillus megaterium* (Liu et al., 2021). The analysis results in Figure 11B indicated that the biostimulation solution successfully activated ureolytic bacteria in the samples.

### 3.7 Mechanism of sand reinforcement by biostimulation

**FIGURE 12:**
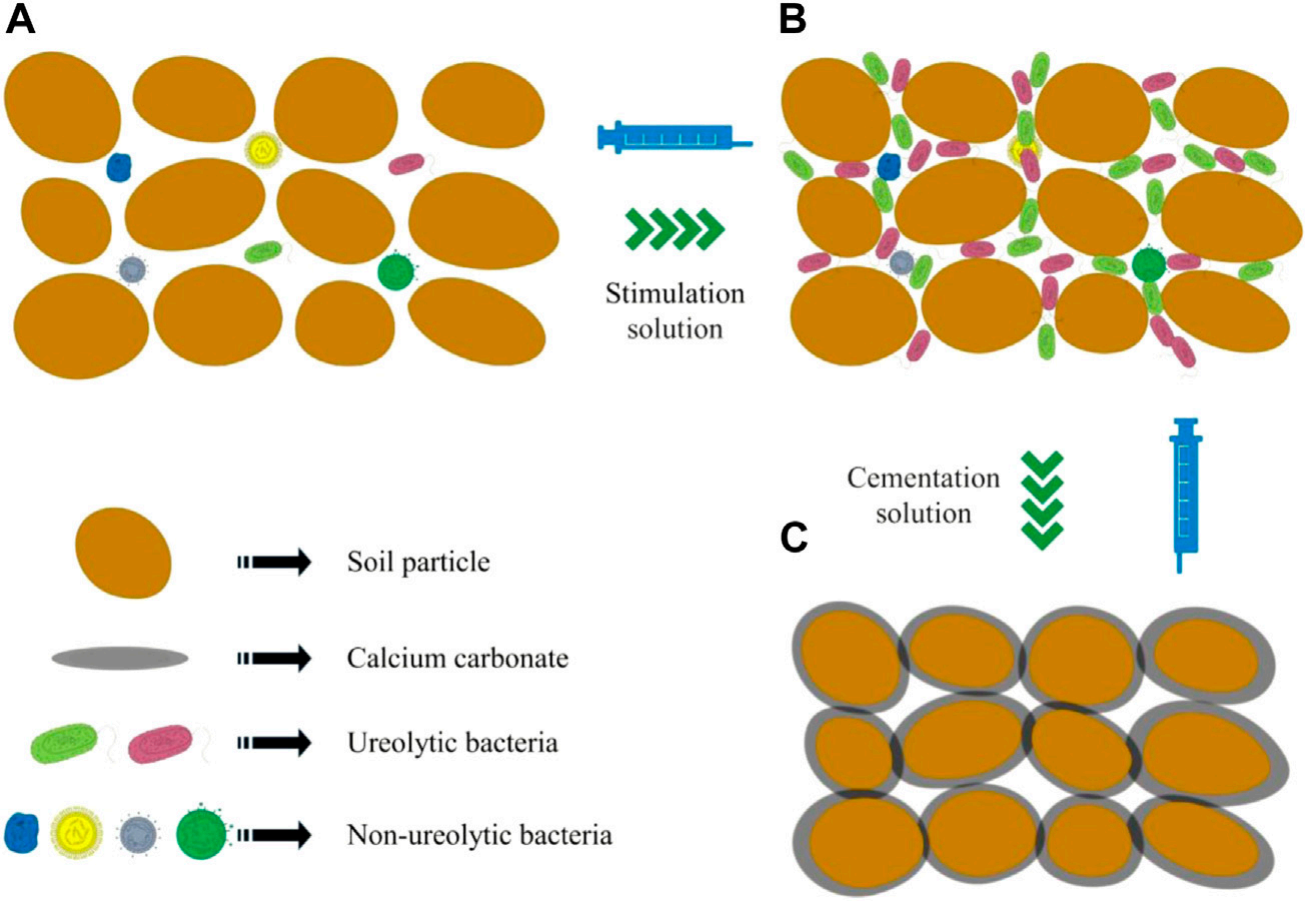
Mechanism of soil reinforcement by stimulation of native microorganisms for biomineralization **(A)**: untreated sample; **(B)** biostimulated sample; **(C)** biomineralized sample).

Ureolytic microorganisms are widely distributed in natural soils (Mahanty et al., 2013). The results of this study confirmed the existence of ureolytic microorganisms in sea sand (Figure 12A). After the sample was injected with a stimulant solution, urease-producing bacteria multiplied and became the dominant strain (Figure 12B). Urea-producing bacteria decompose urea to produce carbonate, and the calcium carbonate precipitated *via* the combination of carbonate and calcium ions binds sand particles together (Figure 12C), thus improving the strength of the samples. As interactions among a variety of bacteria may be involved, the biomineralization process induced by the biostimulation strategy is complex and requires more in-depth studies.

### 3.8 Economic and environmental benefit analysis

At present, the construction and operation of buildings cause 50% of the global carbon dioxide emissions (Achal and Mukherjee, 2015). Among building materials, cement concrete has become the most widely used artificially made material in the world (Imbabi et al., 2012). Although people have realized that the transformation of building materials is essential to ensure sustainability. However, cement production still accounts for about 8% of the global anthropogenic carbon dioxide emissions (Andrew, 2019). Injection of cement slurry or deep mixing with cement is one of the main current soil improvement methods (Bahmani et al., 2014). However, this method relies on artificial materials and mechanical equipment, which means a large amount of carbon dioxide emissions and energy requirements.

Although microbial grouting ($∼7 per m^3^ of soil) seems to be more expensive than chemical grouting ($∼9 per m^3^ of soil), it has relatively little impact on the environment (Mujah et al., 2016). In order to reduce the cost of MICP, some alternatives were proposed. The cost of MICP can be reduced by 70% by using the recovered lactose mother liquor and corn steep liquor as nutrition sources (Achal et al., 2010). Similarly, suitable replacements can be found for the other components of MICP (Cheng et al., 2014).

Bioaugmentation and biostimulation are the two main biotreatment strategies for soil improvement by MICP (Liu et al., 2021). Using indigenous bacteria in the environment for biostimulation to induce mineralization can not only prevent species invasion, but also effectively reduce costs and prevent blockage by eliminating bacterial culturing and injection procedures. Therefore, biostimulation is more economical and environmentally friendly than bioaugmentation (Litchman, 2010).

The cost of MICP treatment depends on the specific process and ingredients used. In addition, because MICP is a relatively new technology, there are few reports on the implementation of large-scale projects, and the actual treatment cost can be difficult to estimate. A quantitative sustainability assessment has been conducted for a simplified hypothetical engineering construction scenario (Rahman et al., 2020). The cost of treatment, together with its environmental benefits in terms of reducing the CO_2_ and energy footprints, has been estimated and compared with conventional construction methods and materials. The results show that MICP treatment brings 19% environmental benefit (CO_2_ emission reduction) and 25% economic benefit.

## 4 Conclusion

Based on biomineralization, a biostimulation strategy optimized by the response surface methodology was proposed to reinforce reclaimed sand. The detailed conclusions are as follows:

(1) The urease activity of the sample optimized by the response surface methodology was 1.17 times higher than that of the sample treated with the initial stimulation solution. The concentrations of molasses, ammonium chloride, nickel chloride, urea and YE in the optimal stimulation solution were 23.21 g/L, 135.48, 0.02, 333 mM, and 0.2 g/L, respectively, and the pH value was 8.64.
(2) A comprehensive consideration of the reinforcement effect and cost showed that using the optimal stimulation solution and 1.0 M cementation solution over 10 cycles is ideal for reinforcing reclaimed sand. The uniaxial compressive strength of the samples reached 3.54 MPa.
(3) SEM results showed that the surfaces of the sand particles were covered with calcium carbonate after mineralization, and the concentration of the cementation solution affected the morphology of the calcium carbonate crystals. XRD analysis results revealed that the final product of microbial mineralization was calcite, which improved the mechanical properties of the samples.
(4) After the samples were treated with the stimulation solution, ureolytic microorganisms became the dominant bacteria in the samples, which is a prerequisite for reinforcing reclaimed sand by biomineralization.

Biostimulation is an effective method to reinforce reclaimed sand; however, the actual application effect requires further examination.

## Data Availability

The data that support the findings of this study are available from the corresponding author, upon reasonable request.
